# How are monospecific stands of invasive trees formed? Spatio-temporal evidence from Douglas fir invasions

**DOI:** 10.1093/aobpla/ply041

**Published:** 2018-07-04

**Authors:** Martin A Nuñez, Juan Paritsis

**Affiliations:** 1 Grupo de Ecología de Invasiones, INIBIOMA, CONICET–Universidad Nacional del Comahue, Bariloche, Argentina; 2 Laboratorio Ecotono, INIBIOMA, CONICET–Universidad Nacional del Comahue, Quintral, Bariloche, Argentina

**Keywords:** Dendrochronology, ectomycorrhizal fungi, monospecific, monotypic, Patagonia, *Pseudotsuga menziesii*

## Abstract

Invasive plant species can produce many impacts on native communities. Impacts can be especially important when the non-natives reach high densities, producing monospecific stands where little grows besides the non-native species. We propose three basic pathways by which monospecific stands of invasive tree species are formed: (i) gradually from the propagule source, (ii) via synchronous establishment and (iii) following several pulses of synchronous establishment. Different patterns can produce different impacts through time and may require different management techniques. This study aims to further our understanding of how monotypic stands of invasive species arise. We documented how monospecific stands are formed during invasion processes by studying patterns of spatio-temporal establishment of several monospecific stands of Douglas fir in Patagonia. We obtained data on tree density, year of establishment, size, distance to the seed source and other related measurements for this tree species along transects from the original seed source (80-year-old plantations) to the edge of the monospecific stand. We found that these monospecific stands arose in a more complex way than expected. While individuals established on average simultaneously over all distances from the seed source, there was substantial variation in time of establishment at all distances. Also, tree density was higher near the source than far from it. Different factors can account for the observed pattern of tree establishment, including seed dispersal, mycorrhizal facilitation and herbivory. Our results elucidate the complexities of spatio-temporal pattern of formation in monospecific stands. This understanding can improve management strategies and techniques for this invasion and other plant invasions in different regions.

## Teaser

Invasive plant species can produce many impacts, especially when they form monospecific stands. However, little is known on how these stands are formed. We propose three pathways for their formation: gradually, via synchronous establishment or following several pulses of synchronous establishment. We documented how monospecific stands are formed during invasion of Douglas fir in Patagonia. We found that these stands arose in a complex way. While individuals established on average simultaneously, there was substantial variation in year of establishment across the stand. Our results help elucidate the mechanisms of stand formation, and may contribute to appropriate management strategies.

## Introduction

Non-native plant species have notable effects on native communities, ranging from changes in fire regimes to changes in soil nutrients, water and light availability, which may alter species dominance and diversity of the native biota (Pyšek *et al.* 2012). Perhaps the most notorious effect is when populations of non-native plants become superabundant and form monospecific stands in which only the non-native species thrive. Monospecific stands are common among invasive plant species and have been described in many different ecosystems. For example, several woody species, grasses and ferns form monodominant stands when invading (Asner and Beatty 1996; Kittelson and Boyd 1997; Portela *et al.* 2009; Cavaleri *et al.* 2014). Once the invasive species has formed a monodominant stand, it becomes extremely difficult to restore the area. This may hold true even after the exotic species has been eradicated, because the legacy of a monodominant stand often results in soil and other key structural alterations (Jordan *et al.* 2008).

**Figure 1.**
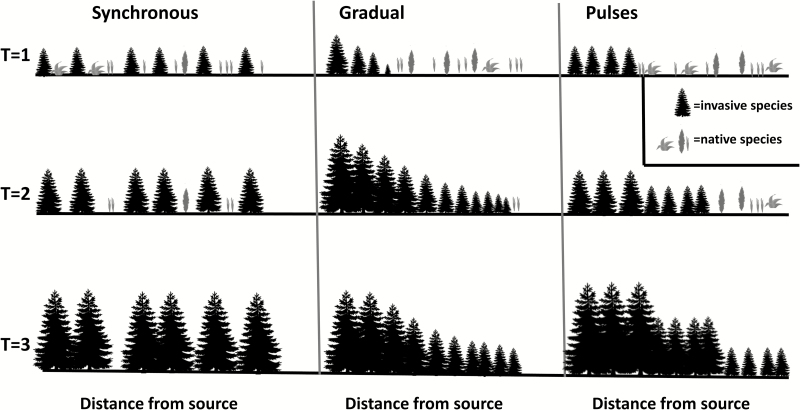
Diagram of possible scenarios of monotypic stand formations.

Despite the large impact and commonness of monospecific stands of non-natives in many ecosystems throughout the world, little is known about how they form. A number of non-mutually exclusive mechanisms are responsible for the superior competitive ability of some invasive non-natives compared with local species. The various mechanisms responsible for creating these areas dominated by non-native species can produce different patterns of stand formation. For invasive species that can exclude or suppress local species, we propose three main ways in which monospecific stands can arise (Fig. 1). One is gradually via vegetative growth or short-distance seed dispersal and establishment that will produce a clear relation between age and distance to source. Another way is synchronous establishment owing to adequate conditions and enough propagule sources over a large area, which will produce an even-aged structure with one clear cohort dominating the invaded area. The last one entails pulses, where the invaded patch grows in several pulses of synchronous establishment, a combination of the previous two pathways. Knowing the patterns of formation of monodominant invading stands can provide valuable information for their management, since different patterns may require different management strategies (Table 1).

**Table 1.** Possible pathways of the formation of monospecific stands and management implications.

Type of monotypic stand formation	Possible mechanisms	Management implications
Synchronous	Climatic window of opportunity for the establishment of the plants. Change in land use. Threshold in levels of herbivory or abundance of mutualisms	Understanding what triggers the phenomena is necessary to prevent (e.g. avoid a type of disturbance) and anticipate it (e.g. if extreme weather events are responsible). Frequent monitoring to detect incipient invasions is fundamental
Gradual	Vegetative reproduction, low long-distance dispersal	Create barriers for their expansion. Frequent removal of invading individuals
Pulse	Combination of mechanisms for gradual and synchronous	Same as synchronous

Multiple studies address the process of stand formation by native tree species. Typically, the formation of monodominant stands of native trees is the consequence of a large-scale disturbance. After a disturbance, formation of the stand could occur as a synchronous establishment, as happens with obligate seeders that have a persistent seed bank (e.g. post-fire *Pinus contorta* establishment) (Anderson and Romme 1991). Alternatively, encroachment can occur gradually or in pulses from remnants at the edge of the disturbed area, as is the case for some post-fire colonizers (e.g. *Nothofagus pumilio*) (Paritsis *et al.* 2015). Other factors, such as changes in disturbance regimes and climate change, are also important triggers for formation of monodominant stands of native trees. For non-native species formation of monodominant stands is not clear, and for invasive conifers it has been proposed to occur as a gradual process from the seed source (e.g. encroaching a similar distance every year from the original source) (Zenni *et al.* 2014; Pauchard *et al.* 2016). Documenting the process of stand formation of non-native trees provides basic information, which allows formulating hypotheses regarding the causal mechanisms. However, it is often difficult and impractical to follow rigorously the formation of an invasive tree stand given the long time intervals involved.

This study aims to contribute to the understanding of the processes of non-native tree stand formation. We conducted this research by analysing Douglas fir invasions in Northwestern Patagonia. We documented the formation of several large monospecific stands of Douglas fir on Isla Victoria, Nahuel Huapi Patagonia, where they are non-native (Simberloff *et al.* 2002). To determine the main spatio-temporal process of stand formation, we obtained years of establishment for the invading trees with dendrochronological techniques along with their spatial location relative to the propagule source (i.e. plantation). We then inferred which pattern of stand formation best matched the observed data and elaborated on what mechanisms may be responsible for the patterns and how such processes should be managed.

## Methods

### Study site

Isla Victoria is an island 20 km long and 4 km across at its widest point located within Nahuel Huapi National Park, Argentina. At the beginning of the 20th century the island began to suffer large human-mediated disturbances that substantially ceased with the creation of the National Park in 1934 (Koutché 1942; APNA 1988). Today most of the island is covered by forests of *Nothofagus dombeyi* and *Austrocedrus chilensis* trees. In 1925, the national government started a tree nursery with different objectives including reforesting the burned areas of the island. More than a hundred species were introduced, many in large numbers (Simberloff *et al.* 2002). Most of them thrived in the island. For example, of the 22 pine species introduced to the island, 19 were well established and producing seeds 20 years after their introduction (Barrett 1952). Two species are successfully invading the native forest in the area (Douglas fir, *Pseudotsuga menziesii*, and common juniper, *Juniperus communis*), with Douglas fir by far the most abundant non-native woody species (Simberloff *et al.* 2002).

### Sample collection

We selected *P. menziesii* plantations that exhibited dense invasion fronts on Isla Victoria. We set up seven transects perpendicular to each plantation’s edge and extended them along the invasion front until *P. menziesii* canopy cover was <50 %. Along each transect, we established sampling plots starting at 2 m from the edge of the plantation (defined as distance 0) and spaced at 10-m intervals. In each plot we selected four *P. menziesii* individuals within a radius of 2 m, two of which had the largest diameter at breast height (dbh = 1.3 m) and the remaining two of which were randomly chosen. Individuals were felled and we collected the basal section of the stem in order to date the year of tree establishment. One cut was conducted at ground level (±5 cm) and the other cut was made 5–10 cm above the first cut. We recorded tree height, dbh, basal diameter and presence of cones for all harvested trees. Additionally, we estimated *P. menziesii* and *N. dombeyi* canopy cover (%) >5 m above the ground, and we recorded number of *P. menziesii* seedlings (i.e. height < 1.4 m), saplings (i.e. height > 1.4 m; dbh < 4 cm) and trees (i.e. dbh > 4 cm) within a 0.8-m^2^ subplot centred within the larger 2-m-radius plot. In the laboratory, stem sections were air-dried and sanded on both cut surfaces. Tree rings on both surfaces were counted under a 10–70× dissection microscope, and the oldest count was kept as the year of establishment. Calendar dates were assigned to rings according to the southern hemisphere tree-ring dating convention, which assigns an annual ring to the calendar year in which the annual ring formation begins (Schulman 1956). In addition to collecting trees along transects, we opportunistically sampled invading trees into the *N. dombeyi* forest outside the dense invasion stands. We identified seven isolated areas with invading *P. menziesii* individuals in the forest 100–220 m from the plantation’s edge, and we set up sampling plots to collect individuals following the same protocol as for sampling plots of randomly chosen individuals along the transects.

To determine the age of the *P. menziesii* source plantations, we collected increment cores at ground level (±10 cm) from five trees per plantation, and we prepared tree-ring samples according to standard dendrochronological procedures (Stokes and Smiley 1968). All tree-ring series were visually crossdated using marker rings and dated. In addition, we obtained documentary records with approximate dates of the plantations’ establishment on Isla Victoria that we used to corroborate the tree-ring dates.

### Data analyses

We used linear regression analysis to evaluate the spatio-temporal dynamics of *P. menziesii* invasion into the *N. dombeyi* forest. First, we regressed year of establishment of the invading individuals in the dense stand (only those that were randomly chosen) on their distance from the edge of the plantation. Then, we ran another regression for the individuals with the largest diameter. Finally, we ran a third regression in which we added the individuals outside the dense invasion stand to the initial regression analysis (i.e. that with randomly chosen individuals within the stand).

We also evaluated the relationship of distance from plantation and basal diameter, and height of the individuals with regression analyses. These regression analyses were carried out separately for the randomly chosen individuals and for those with the largest diameter. We also used linear regression analyses to examine the potential association between distance from the plantation and *P. menziesii* seedling, sapling and tree density, and also canopy cover of *P. menziesii* and the native dominant tree, *N. dombeyi*. Additionally, we conducted correlation analysis between *P. menziesii* and *N. dombeyi* canopy cover.

## Results

### Spatio-temporal dynamics of invasion

**Figure 2.**
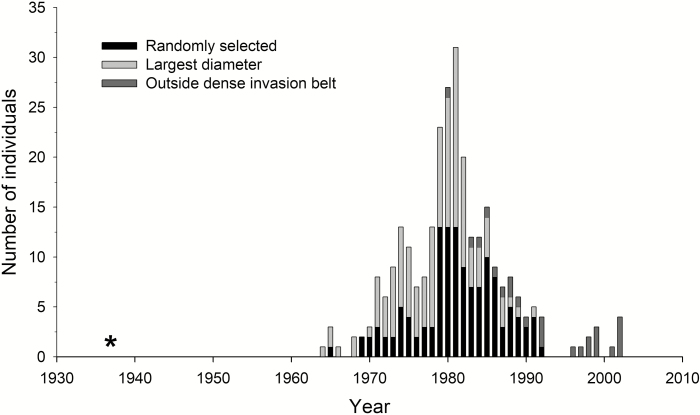
Douglas fir recruitment at Isla Victoria. Bars indicate the number of individuals established each year based on tree-ring analysis. Bar patterns correspond to individuals collected based on three different criteria within the sampling plots: randomly, largest diameter and outside the dense invasion belt. The asterisk indicates approximate year of plantation establishment.

Tree-ring samples show that *P. menziesii* plantations were initiated in 1937.5 ± 2.7 (mean ± SE), and the establishment of the first trees within the invasion belt started by the mid-1960s. Significant invasion, however, started in the early 1970s, ca. 35 years after the plantations were initiated, and showed a steady increase in recruitment until the early 1980s, when rate of tree establishment started decreasing, stopping in the early 1990s. Outside the dense invasion belt, invasion started in the early 1980s (Fig. 2) and is still occurring as indicated by the presence of seedlings in the native forest (Nuñez *et al.* 2008b).

**Figure 3.**
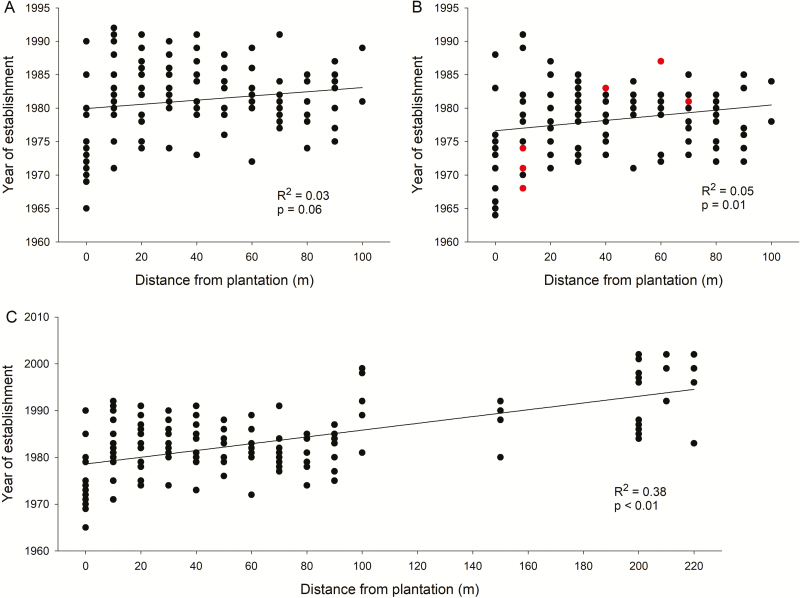
Relationship between distance from the plantation’s edge (seed source) and year of establishment of Douglas fir individuals on Isla Victoria. Three separate regression analyses were conducted: (A) first for randomly chosen individuals within the dense invasion belt, (B) second for individuals with the largest diameter within the dense invasion belt and (C) third for randomly chosen individuals within and outside the invasion belt. Red dots in (B) indicate reproductive individuals (with cones). Regression lines, *R*^2^ and *P* values are indicated on each plot.

Regression analyses showed no relationship between age and distance from plantation of the randomly selected individuals within the monodominant stands (*R*^2^ = 0.03; *P* > 0.05; Fig. 3A). Similarly, the analysis with only the largest individuals also showed no association between age and distance from plantation (*R*^2^ = 0.05; *P* < 0.05; Fig. 3B). There was great variation in establishment year for trees at the same distance from the plantation. Most individuals within the dense invasion belt (75th percentile) at all distances from 0 to 90 m from the plantation’s edge established over a 15-year period, between 1972 and 1987 (Fig. 3A and B). When we included the individuals outside the invasion belt in the regression analysis, we found a positive and significant association between establishment year and distance from plantation (*R*^2^ = 0.37; *P* < 0.05; Fig. 3C).

### Spatial patterns of individual size

**Figure 4.**
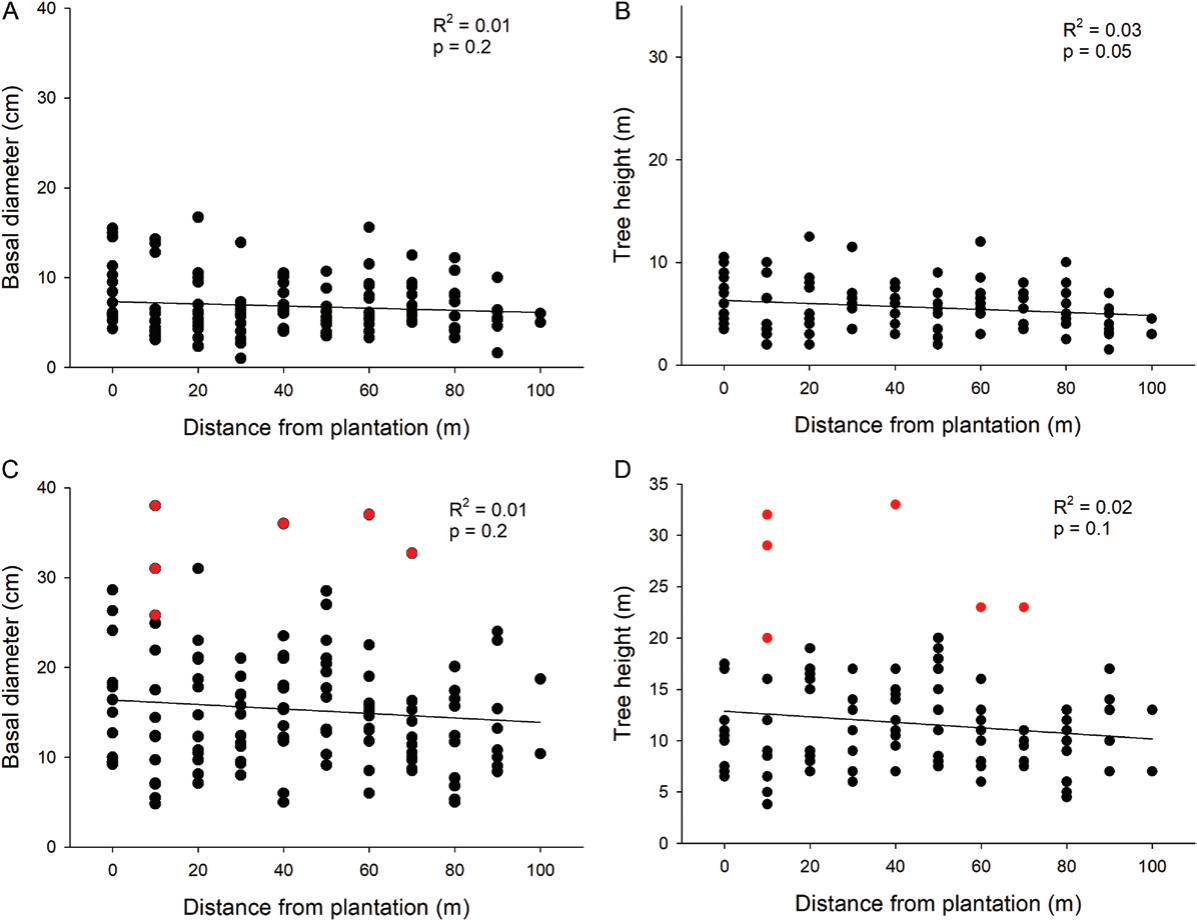
Relationship between distance from the plantation’s edge (seed source), and basal diameter and tree height of randomly chosen individuals (A, B) and individuals with the largest diameter (C, D) of Douglas fir on Isla Victoria. Red dots in (C) and (D) indicate reproductive individuals (with cones). Regression lines, *R*^2^ and *P* values are indicated on each plot. Note the different scale on the y axis in B and D.

Size (basal diameter and height) was not correlated with distance from the plantation in the randomly selected individuals or in the individuals with the largest diameter (Fig. 4). Only six trees of the 257 sampled (both randomly selected and with the largest diameter) in the *P. menziesii* invasion belts adjacent to the plantations were reproductive (i.e. had cones). The six reproductive individuals were among those with the largest basal diameter and were among the tallest sampled trees (Fig. 4C and D). However, individuals with cones were not among the oldest, with establishment dates ranging from 1968 to 1987 (mean ± SE: 1977.5 ± 2.7) (Fig. 3B).

### Spatial patterns of individual density

**Figure 5.**
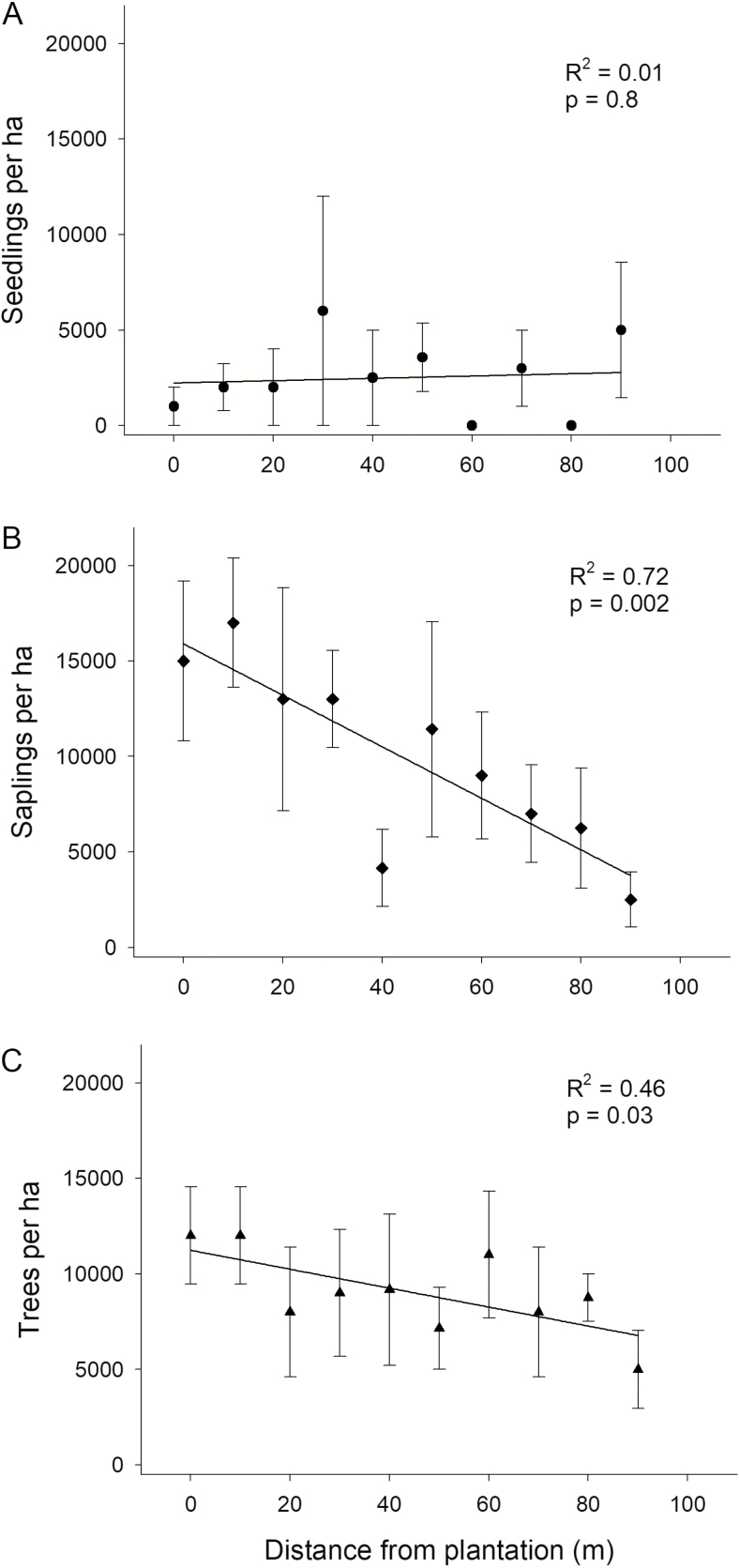
Mean density (±SE) of Douglas fir (A) seedlings, (B) saplings and (C) trees at 10-m intervals from the plantation’s edge. Regression lines, *R*^2^ and *P* values are indicated.

**Figure 6.**
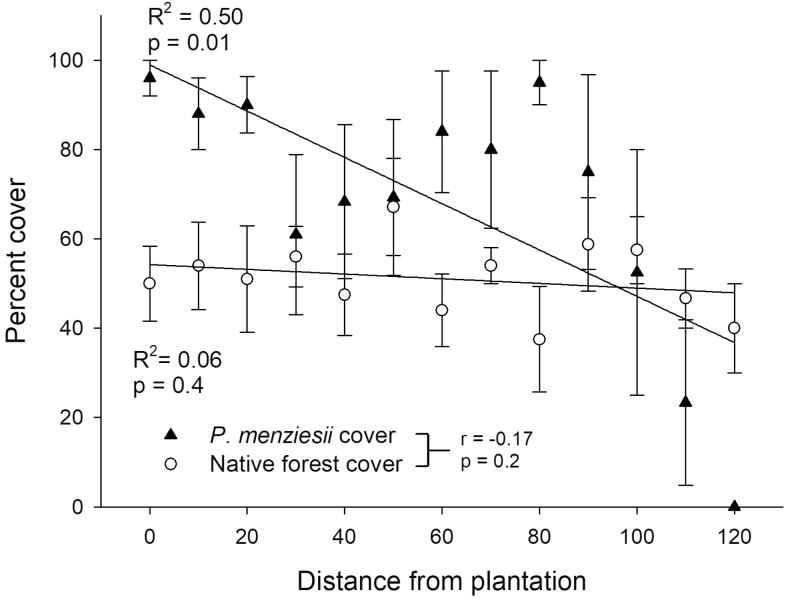
Mean percent canopy cover (±SE) (>5 m height) of native forest species (mainly *Nothofagus dombeyi*) and *Pseudotsuga menziesii* along transects in the invaded area. Regression lines, *R*^2^ and *P* values are indicated. Correlation coefficient and significance between percent canopy cover of *N. dombeyi* and *P. menzesii* are shown after the legend.

*Pseudotsuga menziesii* seedling density within the invasion belt was low compared to that of saplings and trees and showed no association with distance from the plantation’s edge (Fig. 5A). On the other hand, there was a pronounced decline in sapling density (from 15000 ± 4183 at 0 m to 2500 ± 1443 saplings per hectare at 90 m) and tree density (from 12000 ± 2549 at 0 m to 5000 ± 2041 trees per hectare at 90 m) from the edge of the plantation to the limit of the invasion belt (Fig. 5B and C). Although the invasion belt ended abruptly at nearly 80–100 m from the edge of the plantation, there was not an underlying change in the amount of cover of the native forest at these distances. There was a significant negative relationship between distance from plantation and *P. menziesii* cover but no association between *N. dombeyi* canopy cover and distance from the plantation (Fig. 6). Similarly, *P. menziesii* cover and *N. dombeyi* canopy cover were not significantly correlated (Fig. 6).

## Discussion

### Findings

#### Spatio-temporal dynamics of invasion.

Our results highlight the complexity of the formation of a monodominant stand. In some aspects, the formation of the studied monodominant stands occurred synchronously, but in others it followed a more gradual pattern related to location of a source. There was no trend in time with regard to average year of establishment, but most trees in the area occupied by a monodominant stand established over a 15-year period. Therefore, there was not a single establishment event but several events. Also density of trees was lower far from plantations, which highlights the fact that invasion across the stand was not spatially homogeneous. This pattern of stand formation has important implications for tree invasion research as well as for invasion management. One implication is that space-for-time substitutions—commonly used in plant invasion research (Zenni *et al.* 2014; Pauchard *et al.* 2016)—cannot be applied at the stand scale, because all trees in the monodominant stand may be, on average, of similar age, so trees near the plantation’s edge are not necessarily older. Another significant outcome was the timing of invasion onset, which occurred ca. 35 years after plantation establishment and ~15 years after trees within plantations became reproductive. Another complexity is that in the areas beyond the monodominant stands individuals were indeed younger, suggesting the potential pulse or gradual formation of monodominant stands that may occur in the future. The observed spatio-temporal patterns, although more complex than initially hypothesized, serve as an initial and necessary stage towards understanding the process of formation of these monodominant stands and allow the formulation of hypotheses on the potential underlying mechanisms.

Evidence from the current study on Isla Victoria shows that time lags from seed production to invasion onset can be expected for *P. menziesii*. Assuming a full seed production onset of individuals in the plantation at 15–20 years of age (i.e. 1952–57) (Broncano *et al.* 2005), and in light of the fact that the invasion began in the early 1970s, Douglas fir did not invade the area for >15 years after large-scale seed production started. Nevertheless, this species was able to invade massively ca. 35 years after plantation establishment. Seed dispersal from planted trees may be an important factor explaining the observed invasion, but positive feedbacks among introduced trees can also help explain the clear and abrupt pattern of fir dominance. In addition, density-dependence in enemy attacks where seed predators and herbivores avoid areas with high fir densities could also help explain the observed pattern. Preventing firs from reaching the density at which positive feedbacks operate could be key to preventing formation of monospecific stands.

#### Spatial patterns of individuals’ size.

No clear pattern emerged of different basal diameters or heights at different distances from source (i.e. plantations). Also there is no clear spatial pattern of individuals producing seeds. So there is no clear benefit or disadvantage for the trees to be near the plantations in the monodominant stands. Regarding the mature individuals, it was surprising that so few were producing seeds (2.3 % of the total trees had cones). This lack of seed production may be, in part, a reason why the invasion is not advancing more rapidly and has not yet colonized large areas of Isla Victoria (Simberloff *et al.* 2003).

#### Spatial patterns of individuals’ density.

Even though age of establishment and size were unaffected by distance from plantation, densities of trees and saplings were lower far from it. This observation highlights the fact that invasion across the monodominant stand is not spatially homogeneous. This pattern cannot be explained by changes in native tree cover, given that no association was found between native tree cover and distance from the plantation. Factors such as seed dispersal from the original trees may have played a role (Nathan *et al.* 2000). In contrast to the pattern seen in older trees, seedling density was low but homogeneous, suggesting that the current pattern of seedling establishment may differ from the one found while the stand was forming, possibly owing to the current presence of some reproductive trees inside the invaded stand.

### Suggestions for future work

Multiple factors can cause the observed spatio-temporal pattern of tree establishment (Byun *et al.* 2015). However, seed production by invading trees does not seem to be key, since most trees in the invasion front do not produce seeds (unlike trees in plantation, which produce many cones) (Barrett 1952). This may be common or not to other invasions; more research is needed to see how general this pattern is. Disturbance generated in an area could also drive synchronous establishment. However, there is no clear evidence of recent disturbance in our study, and the area is part of a section of the national park with very low human visitation rates and impacts. Also changes in percent cover of *P. menziesii* with distance from plantations cannot be explained by changes in the canopy cover of the dominant native tree, *N. dombeyi*. This observation suggests that establishment of *P. menziesii* was not explained by changes in native tree cover with distance from plantations (Fig. 6).

Why the monodominant patch stopped at around 100 m is an open question. Limitation in seed dispersal distance from the original planted trees can help partially explain this pattern, since density of invading trees was lower far from plantations. However, it does not seem to be the only answer, given the abrupt change in tree abundance relatively far from the source, which may not be explained by the gradual decay in seed dispersal with distance that has been seen in conifer species in their native range (e.g. Nathan *et al.* 2000). In the presence of density-dependent positive feedbacks, where the existence of an established Douglas fir individual facilitates the establishment and survival of another, a well-defined border can be expected, in which low densities of non-native trees make them less able to establish compared to the native species. Presence of mycorrhizal fungi (Nuñez *et al.* 2009; Nuñez and Dickie 2014) and mycorrhizal networks (Teste and Simard 2008; Teste *et al.* 2009) could contribute to explaining this abrupt end of the dense invasion patch. Soil mutualisms can produce a positive effect when there is a high density of trees. This effect can be direct, when the new trees obtain a subsidy from the older trees, as can be expected if mycorrhizal networks are important (Klein *et al.* 2016), or indirect, when production of recalcitrant litter can promote some type of ectomycorrhizal trees (Bever *et al.* 2010). Many monotypic stands around the world are associated with ectomycorrhizal associations, such as those formed by Douglas fir (Bever *et al.* 2010).

Other explanations for the abrupt change at 100 m could involve density-dependent effects of enemies. For example, on Isla Victoria, herbivory by non-native deer and seed predation by native rodents and birds has been proposed as a factor explaining the low levels of invasion observed in many areas of the island (Nuñez *et al.* 2008a, 2008b; Relva *et al.* 2010). Herbivores and seed predators could avoid areas where *P. menziesii* is abundant owing to lack of preferred habitat (as in the case of seed predators; Nuñez *et al* 2008b) or lack of preferred food items (as in the case of deer; Nuñez *et al* 2008a).

### Concluding remarks

This study suggests that documenting the spatio-temporal pattern of formation of *P. menziesii* monodominant stands is key to understanding the invasion process and to improving management strategies. Describing the patterns of formation of monodominant stands of non-native species is critical to generate informed hypotheses aimed at understanding the mechanisms behind invasion. From a management perspective, different patterns of formation require different management strategies (Table 1). The impact of isolated individuals of any non-native species is likely to be low, but the impacts of ultra-dense populations of any species are likely to be high. Therefore, the study of the formation of monodominant stands can be crucial to reduce the impact of non-native populations.

## Sources of Funding

We thank NSF (DEB 948930) of the USA, and Biosilva (#5), PICT (2016-1421) and PICT (2014-0662) of Argentina.

## Contributions by the Authors

M.A.N. and J.P. designed the study, organized the field work, analysed the data and wrote the manuscript.

## Conflict of Interest

None declared.
